# Living with Uncertainty: Acting in the Best Interests of Women

**DOI:** 10.1155/2012/524936

**Published:** 2012-11-01

**Authors:** Erica Gollub, Zena Stein

**Affiliations:** ^1^Department of Epidemiology, Robert Stempel College of Public Health and Social Work, Florida International University, 11200 SW 8th Street, Miami, FL 33199, USA; ^2^Department of Epidemiology, Mailman School of Public Health, Columbia University and HIV Center for Clinical and Behavioral Studies, New York State Psychiatric Institute, New York, NY 10032, USA

## Abstract

A recent multi-country study on hormonal contraceptives (HC) and HIV acquisition and transmission among African HIV-serodiscordant couples reported a statistically significant doubling of risk for HIV acquisition among women as well as transmission from women to men for injectable contraceptives. Together with a prior cohort study on African women seeking health services, these data are the strongest yet to appear on the HC-HIV risk. This paper will briefly review the Heffron study strengths and relevant biological and epidemiologic evidence; address the futility of further trials; and propose instead an alternative framework for next steps. The weight of the evidence calls for a discontinuation of progestin-dominant methods. We propose here five types of productive activities: (1) scaling injectable hormones down and out of the contraceptive mix; (2) strengthening and introducing public health strategies with proven potential to reduce HIV spread; (3) providing maximal choice to reduce unplanned pregnancy, starting with quality sexuality education through to safe abortion access; (4) expanding provider training, end-user counseling and access to male and female barriers, with a special renewed focus on female condom; (5) initiating a serious research agenda to determine anti-STI/HIV potential of the contraceptive cervical cap. Trusting women to make informed choices is critical to achieve real progress in dual protection.

## 1. Introduction

The recent Heffron et al. [1] multi-country study on hormonal contraceptives (HC) and HIV acquisition and transmission among African HIV-serodiscordant couples reported a statistically significant doubling of risk for HIV acquisition among women as well as transmission from women to men for injectable contraceptives. A more modest effect in the same direction was was also reported for combination oral contraceptives (COCs), although not attaining statistical significance. This study, together with Morrison et al.'s analyses [2, 3] on a cohort of African women seeking health services, represents the strongest data yet to appear on the association. It is unlikely that other study designs, including a randomized controlled trial (RCT), could provide stronger evidence for causal inference. Therefore goes our argument: these studies provide the weight of the evidence and the best we can get for the next decade. 

Of course, one study, however good, cannot make for certainty. But by now there have been over 50 published papers and several comprehensive reviews, emphasizing the different study populations and analytic strategies and designs, all mainly emanating from high prevalence sites. Overall, the results for DMPA have shown at least a modest risk for HIV, not necessarily statistically significant depending mainly on sample size. Studies not indicating risk often have serious methodologic shortcomings, such as infrequent timing of exposure and outcome ascertainment [4], poor statistical power [5], or aberrant comparison groups [6]. 

However, Morrison et al., the author of the editorial accompanying the Lancet issue that published Heffron et al., and who has published other major works in this field, particularly emphasized the additional risks among young women. A recent study from South Africa demonstrated a raised risk of DMPA that only marginally failed to reach statistical significance and suggested that NET-EN, the alternative injectable contraceptive used especially by young women in South Africa, might also be a risk factor though of lesser magnitude [7]; a second South African study also found a significant association of DMPA with chlamydia infection [8]. Neither the recent CROI nor microbicide meetings, in which these relationships were intensively discussed, showed any data to challenge seriously the relationships outlined here. The immunosuppressive effects of DMPA may bring other health risks that are particularly pronounced among young women. For example, recent data have indicated a doubling of risk for breast cancer in women aged 20–44 years associated with recent or longer term (>1 yr) use of this method [9]. 

This article will cover four issues: first, review the strengths of the Heffron study; second, reexamine and update biological and epidemiologic evidence to support the HC-HIV association; third, address the likely futility of further trials on this association; and finally propose an alternative framework for next steps. 

## 2. Epidemiological Strengths of Heffron

Heffron et al. addressed a large and vulnerable high-risk population. The study followed close to 4000 HIV-serodiscordant couples across seven African countries for which HSV-2 infection status was known for each partner. Importantly, timing of the acquired HIV infections was tightly linked to the exposure period, a problem in numerous prior studies. The analysis excluded infections among men from an outside partner, via genetic testing of the HIV-1 virus, thus assuring the firmest possible control over HIV exposure source. Contraceptive exposure information was updated quarterly; self-report was validated and study sites often were the providers of the contraceptive methods (personal communication, Helen Rees). Finally, participant retention was high (over 90% at 12 months; over 84% at 24 months), minimizing threat of selective loss-to-follow-up. These four study aspects lent great strength of inference to the observed association. Study aspects leading to weaker causal inference included self-report as the source of information on condom use and sexual frequency. Nevertheless, superior methods for collecting or validating information on these exposures in such a study setting are not possible. The sexual behavior variables were controlled in numerous analytical approaches. Adjustment in Heffron strengthened the observed effect estimate and narrowed the confidence intervals. In Heffron, as in the Morrison analyses preceding, multiple statistical approaches undertaken to control effects of these confounders, moving from less rigorous to more rigorous approaches, did not undermine the observed effect and, as also in Morrison et al., demonstrated a strengthening of the effect estimate. Heffron and coauthors recently presented sensitivity analyses [10] demonstrating a persistence of the effect on HIV risk found in their original analysis. Of note, when restricting the analysis to consistent DMPA-users only (i.e. excluding South African women who also use Net-En), the risk (hazard ratio) climbed substantially, to 3.39 (CI 1.38–11.22). Also of interest, and found in other data sets, COCs, containing a lower dose of progestin, showed weaker effects on HIV risk (see also biology, below). Alternate explanations for this result, that entirely exclude an actual biological effect, are difficult to formulate. 

Finally, that Heffron et al. represented a secondary analysis has been repeatedly emphasized as an important shortcoming; yet this would not appear to detract significantly from epidemiologic inference. Certainly, the number of hormonal contraceptive users was smaller than in a study explicitly designed to assess this association, resulting in relatively wide confidence intervals. Nevertheless, concern that the Heffron analysis, due to its secondary nature, demonstrated “chance findings” is unwarranted. The likelihood that there is *no* underlying effect of HC on HIV risk, when posed against the substantial body of biologically coherent data and epidemiologic evidence in favor of this association generated over 2 decades [11], is vanishingly small. 

## 3. Supportive Biological Evidence and Coherence 

There is no dearth of accrued biological evidence supportive of an increased risk of HIV infection due to endogenous or synthetic progesterone exposure; indeed, numerous biological mechanisms might act synergistically. Marx and colleagues in 1986 in macaques [12] demonstrated increased dramatic thinning of vaginal lining as well as susceptibility to SHIV with progestin treatment. Indeed, progesterone treatment is routinely used in experimental animal protocols to achieve infection. Effects of progesterone to explain the observed role in infection of monkeys include vaginal thinning and loss of keratinization of the epithelial layer, but remain less clear. In humans, Mauck and colleagues [13] found only a modest amount of epithelial thinning in a small series of American women administered DMPA, inconsistent with the degree of thinning seen in Marx et al., thereby weakening a biological plausibility argument through analogy with the monkey data. But recent data from Vishwanathan and colleagues [14] demonstrate SHIV infection risk is increased with only a modestly higher level of progestin, and a small degree of thinning that accompanies the progesterone-dominant phases of the menstrual cycle of pigtail macaques. This study has challenged the argument for vaginal thinning as one mediator of HIV infection. 

Other potential mechanisms to explain an increase in infection risk for women using HC include, on a clinical level, cervical ectopy (ectopy is typically associated with immaturity; Morrison et al. [2, 3] found HIV risk with use of DMPA more marked among young women but a recent study did not support the association [15]), or mediating effects of genital tract infection, and related impact and role of *Lactobacillus* colonization of the vagina; and on a cellular level, cervicovaginal inflammation and viral replication [16]. One study of high-risk women demonstrated that HC users were more likely than non-users to become simultaneously infected with more than one variant of HIV-1 [17, 18]. Kumwenda and colleagues [19] who measured HIV viral load quarterly among women attending general reproductive health services in Malawi, demonstrated both a strong association of DMPA with seroconversion, as well as an interaction of DMPA use with increased viral load around the time of acute infection. No association of DMPA was seen for disease progression. The findings accented the critical nature of the timing of ascertainment of exposure to detection of any underlying HC-HIV association.

Hel and colleagues [20] have extensively reviewed the multiple immunoregulatory effects of progesterone. Estrogen treatment is noted to protect macaques against infection; thus providing some biological coherence to the observation in numerous epidemiologic studies, that COCs are associated with a lower risk of HIV acquisition in women, as compared with DMPA, even when controlling for sexual behavior [3, 7, 16, 21]. Progesterone treatment in some studies increases frequency of Langerhans cells in the vaginal epithelium, an important target cell in HIV early infection events. Chandra et al. [22] recently reported significant increases in vaginal T cells, activation markers, and HIV-1 receptors among healthy women measured at 12 weeks following DMPA administration. Estrogen treatment is associated with a decreased frequency of these cells [20]. Other data converge to strongly support the contention that physiological changes in progesterone exposure during the menstrual cycle account for large differences in infection risk [23]. 

## 4. Is a Randomized Controlled Trial (RCT) Called for and Feasible?

There have been calls for an RCT to strengthen epidemiological inference on HC-HIV risk [24]. It is unlikely that an RCT, however well-designed, would answer the shortcomings of the present, large, observational data set. The main gaps in Heffron et al., confirmation of condom use and coital frequency, are difficult to fill.

First, it is unlikely that any single RCT could replicate or improve on the population diversity represented in the observational data. Exposure interactions with variables such as high-risk sexual behavior, concurrent STI, vaginal ecology variables, and other covariates, all of which may be important in the risk equation, could be entirely missed in the narrow select population of any RCT. Women who agree to be randomized to a contraceptive method (e.g., Depo Provera injection versus IUD) may be different in some important ways among women who would and would not be recruited, limiting the chances to detect an underlying association. Clinical inclusion/exclusion criteria will lead to further homogeneity of the population. Second, it is unlikely that the study could blind women to their assigned method, thereby raising the risk of differential condom use and other sexual behaviors (e.g., sexual frequency) in response to the method's secondary effects or perceived method attributes or efficacy. This undermines a primary rationale for trial design. Such behaviors will need to be captured by self-report, as with observational design; self-report of behaviors may thereby be differential by trial arm. Third, method side effects (such as nausea, breast tenderness, and intermittent bleeding for DMPA), historically a primary cause of discontinuation, would not be altered by trial design. Discontinuation, method switching, and/or poor adherence would reduce the statistical power of the trial to identify and quantify risks with HC, and is a serious threat to trial success. These considerations imply that a trial carries a substantial likelihood of missing an actual, underlying difference in HIV risk with hormonal methods, with the attendant false reassurance of safety. Adherence has been a serious challenge in other large trials of FP methods. Fourth, the assignment of trial arms will certainly be a conundrum. IUDs may pose their own risks of increased STI/HIV; concerns regarding PID are still current (see below). Comparing different hormonal products with each other will require a sample size that may be unattainable and certainly an outstanding resource commitment. Absence of a condom arm would hinder inference considerably; if hormonal and IUD arms are equivalent in HIV risk, what shall we conclude about the relative risk for women using condoms? Yet, randomizing women to nonhormonal/non-IUD contraception reproduces the potential for unequal use of condoms across arms, as mentioned above. Also, ethical arguments have been expressed in relation to randomizing women to a male condom-only condition where partner cooperation is required and often not realized [25].

In short, a RCT would be unlikely to yield greater insights into this issue, but would cost us another better part of a decade to design, conduct, and analyze, during which time HIV incidence would continue to climb, and FP program adaptation would be delayed. The reflexive move toward an RCT must be resisted; theoretical gains of randomized design would not be achieved in practice, as outlined above. The momentum of the scale-up of DMPA in FP programs over the past decade must also be considered in the risk equation and timeline to response—the yearly cost in HIV infections secondary to use of injectables has not yet reached its apex, placing an even greater urgency on decisions to change policy and discontinue use of this contraceptive method. Further, the data in Heffron pointed to a doubling of the risk of HIV transmission to the male partner with use of DMPA. This is the first such report and although not confirmed elsewhere, provides additional arguments for moving this method out of the contraceptive mix as quickly as possible. 

A change in paradigm favoring the scale-up of safer, health-preserving contraceptives to men and women in low-resource, high-HIV risk settings brings clear benefits for generations to come. By contrast the marginal gains to be had in squandering precious resources on a further trial of DMPA, even with the highly optimistic and unlikely assumption that such a trial will be “perfect” and unimpeachable, are small. The move to safer contraceptives is inevitable, as is the need to decide policy in a state of scientific uncertainty. 

## 5. Next Steps: Living with Uncertainty, Acting in Best Interests of Women

Taken together, the biological evidence and epidemiologic evidence from Heffron et al., and Morrison et al. while not definitively ending the debate on whether HCs raise risk of HIV, have nevertheless tipped the scale considerably in favor of a presumption of risk, especially for progestin-dominant injectables. It is no longer ethically justifiable to “stay the course” with current FP programming; the weight of the evidence supports a move away from promotion of progestin-dependent methods. 

This altered course, then, calls for at least five different types of activities, of which our main focus for this article shall be on the last two. 

*Scaling injectable hormones down and out of the contraceptive mix and bolstering provider training as well as counseling activities to support full disclosure policies for users on risks with these methods.* Greatly increased emphasis of educational approaches, and their evaluation at a range of sites will be needed. It must be explained to all concerned—health workers, women at risk, and their partners—that use of hormonal methods, especially DMPA, likely increases women's risk of HIV acquisition. Women cannot be denied this information and the autonomy to act in their best interests as they see it. Protecting women “from themselves” will not advance a women's health agenda. In our view, the recent WHO Technical Statement [26] downplays risks with these products by urging no effective change in policy while at the same time vigorously promoting use of added protection against HIV; it is an unclear message that is hard to decipher for women users. Women should be dissuaded from initiating use of injectables and informed about the reasons and concerns; whether in low or high HIV-prevalence areas, women's right to the information on risk and their ability to exercise choice must be paramount. Counseling should also cite a possible elevated HIV risk for men though stressing this is unconfirmed and carefully refocusing responsibility on the male partner to use condoms for couple protection. 

*Strengthening and introducing public health strategies with proven potential to disrupt HIV transmission,* including universal test-and-treat, treatment upon first diagnosis, circumcision programs, pre- and post-exposure prophylaxis, whether pills or microbicides, and treatment as prevention (Truvada). Addressing implementation strategies for combination prevention is likely to yield large gains in reducing HIV spread [27]. These will lower community/network infection prevalence and thus reduce the impact of contraceptive method interactions with HIV exposure.

*Providing maximal choice to reduce unplanned pregnancy to a minimum, from quality sexuality education to diverse contraceptive methods to emergency contraception and safe abortion access. Scale-up and promotion of the widest range of contraceptive methods to address different age groups and diverse partnership contexts should include permanent methods (male and female sterilization) lowest dose non-progestogen-based hormonals, IUDs (with screening and triaging of users), and male and female condoms—all with careful counseling regarding risks and benefits and need for consistent use for coitally dependent methods.* Current contraceptive choice in high-HIV prevalence areas is limited and must now urgently diversify towards methods that do not increase STI/HIV risk. Women must be informed that all nonbarrier contraceptives involve some level of risk (besides contraceptive failure); and that male and female condoms do not pose these safety risks. Consistent use is key for coitally-dependent methods and pills. Women should be full partners in this choice. 

IUDs are highly effective and do not involve systemic exposure to progestogens, with their immunosuppressive effects [28]. Nevertheless, data remain scarce on possible effects on risk of HIV acquisition. The evidence on PID risk is still worrisome for high-STI prevalence areas [29]. Several authors have offered careful approaches to be used even in such settings, including, first and foremost, commitment to quality of care [30], as well as the use of conservative algorithms to determine candidacy for this method and presumptive antibiotic treatment for women at highest risk [30, 31]. Women users should be educated about PID—the risks and signs and symptoms. 

*Expand provider training, end-user counseling and access to male and female barriers—female condoms, diaphragms, and cervical caps—for sole use, dual use, or multipurpose use, with renewed focus on hierarchical counseling and use of proven interventions to ensure optimal uptake.* Male condom use is certainly efficacious, and must continue to be urged; yet, the enormous obstacles many women face in negotiating their use with partners remain as valid today as they were at the start of the HIV epidemic [32]. Though progress has been made in male condom use rates, a majority of high-prevalence countries report lack of protection at last encounter [33]. Indeed, promoting the male condom now as a satisfactory solution to women's increased risk of HIV with injectable hormones only invites a cynical reception and the planting of the seeds of mistrust in women due to its seeming head-in-the-sand nature, and limited usefulness “on the ground”. Cates and Steiner [34], in an early paper have argued that promoting condoms only for disease prevention may stigmatize the method. Indeed, from a woman's perspective, removing the “contraceptive justification” for male condom use from the argument repertoire—for example, where male partners are aware of hormonal contraceptive use—clearly impacts on likelihood of partner agreement. Thus, male condoms as the second (dual) method is likely to appeal only to highly select population groups such as HIV sero-discordant couples—those keenly aware of their status and mutually committed to protection. For others, recourse to female protection methods is probably still the most promising route. These arguments together point to the need to expand—in name and in deed—access to female methods as dual methods. Proactive promotion of female barriers alongside hormonal methods must start with specific mention of such methods in policy or counseling texts. These safe methods could be promoted both as alternatives to hormonal methods, or to be used in tandem with them—“offset options”—to mitigate against risk of infection while bolstering protection against unplanned pregnancy. Using “hierarchical” strategies [35], the female condom *and* male condom should be uniformly counseled on as equally protective against STI/HIV [36], with cervical barriers (diaphragms, cervical caps, cups, etc.) promoted as “next best”; some of these latter should provide some protection against disease (those infecting the cervix mainly or solely) but less than either of the two condoms. Any serious program of scale-up of female barriers would also require provider training (across FP-HIV-reproductive health (RH) facilities) and positive re-orientation of FP personnel, at all levels, as this has been a serious hindrance to use historically [37–40]. Recent evidence from a South African female condom trial quantifies the substantial reduction in method problems experienced by the user when adequate user support is provided in the initial adoption period [41]. There has been a tendency to discount female barrier methods as impractical and unacceptable though no wide-scale evidence in developing countries supports this contention and indeed there is evidence to the contrary [42–48]. Certainly the existing evidence is wholly insufficient to glean women's actual preferences when given real choice, in an appropriately-designed research study or demonstration program. Involvement of communities of women users of contraceptive services, and their partners, will help inform the most culturally appropriate approaches [40]. Women have potentially more control over female condom use but its impact will be realized only with strong promotional programs and quality counseling including outreach activities to men wherever possible [49], as well as easy access to continuing supplies, to ensure adoption and maintenance of the behavior [36]. 

*Initiation of a serious research agenda to determine anti-STI/HIV efficacy and effectiveness of an important yet overlooked contraceptive method: the cervical cap. Covering the cervix may help reduce HIV risk via: (a) reducing coincidence of cervical inflammation-HIV contact, (b) blocking sperm-mediated HIV infection, and (c) among young women (with immature cervices) for which injectables may carry a very high increased risk of HIV infection.* Advocacy for women-controlled protection has spawned numerous, exciting directions aimed at expanding chemical and physical protection methods for women. Among these, the movement for multipurpose prevention technologies (MPTs) has as its goal to develop products with at least two indications (contraception, STI prevention, HIV prevention, reproductive health enhancement) [50]. New cervical barriers under this rubric include the Silcs diaphragm and tenofovir-releasing rings [51]. Concurrently with development of new methods, however, research on conventional, time-tested and approved female barriers must also proceed with urgency. As one example, there is a clear need for a RCT to address anti-HIV efficacy and effectiveness of the cervical cap, and specify optimal conditions of use as a contraceptive. Although the contraceptive diaphragm has been the subject of a large-scale trial for efficacy in HIV/STI prevention [52] (failing to produce evidence of effectiveness), the cervical cap has so far been passed over, despite the fact that the device may offer distinct advantages over other devices now under study and in development. 

### 5.1. Biological Justification for Cervical Barrier Methods in Women's HIV Prevention

HIV infection across the vaginal wall, as compared to ecto or endocervical transmission—is thought by many to account for most sexually transmitted infections in women due to the large difference in surface area [23]. Nevertheless, considerable questions remain. In particular, due to the possibility of sperm-mediated transport of HIV, uterine peristalsis aiding infection of the upper reproductive tract, and the high prevalence of cervicitis in the developing world, a clear role for cervical barriers in reducing risk of HIV infection certainly exists [53]. Hormonal mediation of HIV receptor cells differs considerably when comparing upper and lower reproductive tracts [23, 54]. Protecting the cervix from inflammation due to infection or trauma, or reduced innate immunity with exogenous hormonal exposure or menstrual cycle stage should still be of high interest for reducing risk of HIV. 

### 5.2. Cervical Caps—Efficacy: Mode of Action

**Figure 1:**
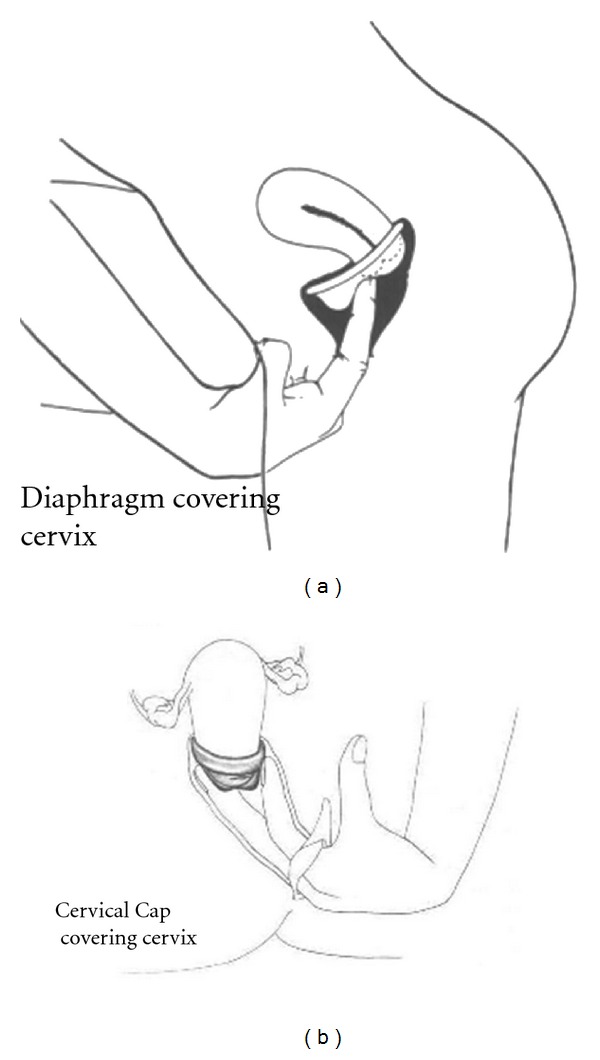
Diaphragm versus cervical cap placement. Courtesy of Rebecca Chalker, *The Complete Cervical Cap Guide*, copyright 1987.

Cervical caps have a distinctly different mechanism of protection than the diaphragm and other “loose” cervical barriers. Like the contraceptive diaphragm, the cap covers the cervix, but the shape of the cap is conical, like the cervix, and the device adheres via gentle suction, rendering the level of protection to sperm and pathogens theoretically much greater than that of the diaphragm, because the cap completely surrounds and encloses the cervix (see Figure 1). The contraceptive efficacy of cap versus diaphragm, in the two available trials, indicates a similar level of protection against unplanned pregnancy [55]; but some of this protection in the case of the diaphragm is likely owing to simultaneous use of spermicide. Much remains to be determined regarding actual use-effectiveness of the cap as a contraceptive or for disease prophylaxis in populations needing dual protection.

### 5.3. Cervical Caps—Effectiveness: User Aspects

**Figure 2:**
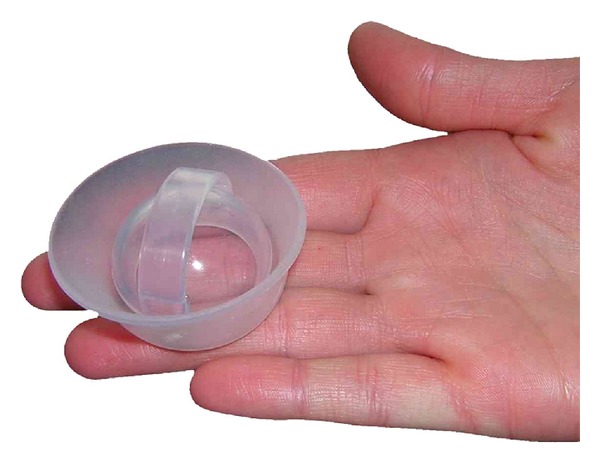
Femcap (courtesy of Alfred Shihata).

 From the user's perspective, there are a great number of advantages to this particular barrier [56, 57]. It can be used without the partners' knowledge, inserted long before sex, and can be used with the male condom. The cap should be compatible with all forms of contraception and could be theoretically quite important in offsetting increased risk of STI/HIV acquisition with hormonal contraceptives or IUDs if used concurrently. The cap may be used for multiple acts without reapplication of spermicide or removal, at a continuous high level of contraceptive efficacy for at least 48 hours, according to present FDA labeling; published data indicate longer wear is possible without safety concerns [58]. Cap devices may be adapted for microbicide release [59]. These devices are in general small, simple, and inexpensive to manufacture, as well as being highly portable, durable, and resistant to temperature and other environmental conditions. The Femcap, as the only currently approved and marketed cervical cap, is made of silicone, thus maximally resistant with no need for replacement over many years [60, 61] (Figure 2).

The user advantages above point to potentially large gains in effectiveness over other cervical barriers when used as an anti-STI/HIV method. The limited field experience with the device in developing country populations suggests acceptability is sufficiently good for larger trials [62, 63]. Evidence from other hard-to-reach populations such as substance-using women suggests the same [64]. An energetic research agenda could bring large STI/HIV prevention gains to potentially diverse populations of women in need. Yet poor global promotion of this device has led to low clinician familiarity, negative attitudes, and consequent low awareness and demand among women [60, 65]. The cervical cap needs an influential champion. 

## 6. Conclusion

There is substantial lack of incentive to altering current FP policy, due to real concerns about the risks of pregnancy for women and children, especially in resource-poor countries. DMPA, as a long-acting, highly promoted, popular contraceptive for women, has a clear public health advantage that extends beyond reducing unplanned pregnancy rates to the reduction of maternal mortality and vertical transmission of HIV infection. Filling the void left by discontinuation of this method will be neither convenient nor simple. Nevertheless, we cannot let the difficulties be an obstacle to change. Numerous other contraceptive options exist and must be deployed with enthusiastic provider support. The price couples pay for contraception need not be HIV infection. The recently-confirmed increased HIV risk with DMPA use has thrown into bold relief our slow progress at entrusting women with a meaningful range of choices in contraception that would also protect them from disease. Increased expectations of women to make up the gap in new HIV infections acquired due to DMPA, by somehow suddenly exerting an extraordinary level of control over male condom use, places the burden squarely on women rather than health systems to adapt and is not a realistic solution. Greater emphasis must be given to full frank informational counseling, and expanded options that women may learn and practice autonomously to reduce HIV risk concurrently with practice of contraception. A change in direction could also promote real bridging and integration across RH, HIV, and FP “camps”, thus opening up new opportunities to better serve women and couples in our public health mission. Finally, the renewed focus on female barrier methods should prompt an accelerated research agenda and important step-ups in funding, both for new technologies we have started to explore, as well as conventional barriers we have yet to exploit. Culling and generalizing proven, effective approaches to female condom promotion remains well within our global capabilities and as yet unrealized. An additional technology with the potential to reduce sexual HIV risk, so far neglected, but calling for revival and an invigorated research campaign, is the cervical cap.
